# The Cumulative Perioperative Model: Predicting 30-Day Mortality in Abdominal Surgery Cancer Patients

**DOI:** 10.31487/j.jso.2020.01.10

**Published:** 2020-03-10

**Authors:** Risa B Myers, Joseph R Ruiz, Christopher M Jermaine, Joseph L Nates

**Affiliations:** 1 Department of Computer Science, Rice University, Texas, USA; 2 Children’s Environmental Health Initiative, Rice University, Texas, USA; 3 Department of Anesthesiology and Perioperative Medicine, Division of Anesthesiology and Critical Care, University of Texas MD Anderson Cancer Center, Texas, USA; 4 Department of Critical Care, Division of Anesthesiology and Critical Care, University of Texas MD Anderson Cancer Center, Texas, USA

**Keywords:** Regression analysis, perioperative period, mortality, decision support techniques

## Abstract

**Objectives:**

1) To develop a cumulative perioperative model (CPM) using the hospital clinical course of abdominal surgery cancer patients that predicts 30 and 90-day mortality risk; 2) To compare the predictive ability of this model to ten existing other models.

**Materials and Methods:**

We constructed a multivariate logistic regression model of 30 (90)-day mortality, which occurred in 106 (290) of the cases, using 13,877 major abdominal surgical cases performed at the University of Texas MD Anderson Cancer Center from January 2007 to March 2014. The model includes race, starting location (home, inpatient ward, intensive care unit or emergency center), Charlson Comorbidity Index, emergency status, ASA-PS classification, procedure, surgical Apgar score, destination after surgery (hospital ward location) and delayed intensive care unit admit within six days. We computed and compared the model mortality prediction ability (C-statistic) as we accumulated features over time.

**Results:**

We were able to predict 30 (90)-day mortality with C-statistics from 0.70 (0.71) initially to 0.87 (0.84) within six days postoperatively.

**Conclusion:**

We achieved a high level of model discrimination. The CPM enables a continuous cumulative assessment of the patient’s mortality risk, which could then be used as a decision support aid regarding patient care and treatment, potentially resulting in improved outcomes, decreased costs and more informed decisions.

## Introduction

There has been an increase in the amount of digital information stored in electronic health records associated with each patient encounter [1]. Typically, outcomes have been associated with data at one time point. To improve on this, we looked at a patient’s hospital surgical course over several time points, reflecting an ongoing clinical picture. Administrative data (such as age, gender, and race) provides a starting point [2]. With major elective surgeries, more information is available preoperatively. Two common comorbidity scores adjusting preoperative risk, beneficial in predicting 30-day complications in surgical patients are the Charlson Comorbidity Index (CCI) and the American Society of Anesthesiology Physical Status (ASA-PS) [3–11]. Together, the CCI and ASA-PS have shown to be useful in predicting complications [12, 13].

The Surgical Apgar Score (SAS) was developed and validated as a simple objective assessment of a patient’s postoperative condition and has been shown to correlate with adverse outcomes [14–18]. The postoperative destination [home, hospital ward or Intensive Care Unit (ICU)] is usually determined preoperatively and has not been well studied as a possible risk factor for outcomes. Furthermore, delayed ICU admission indicates an increased level of care is needed and could be inferred to be associated with poor outcomes. Numerous other specialized and general scores have been developed that predict morbidity and mortality at varying points in the future for different subsets of patients.

We developed a Cumulative Perioperative Model (CPM) that incorporates patient information available as the patient received perioperative care. The CPM predicts 30 and 90-day mortality in cancer patients undergoing abdominal surgery. We follow the patients throughout their clinical course and update the prediction at each logical time step. We use cumulative, easily calculated features based on available knowledge to predict mortality. We compared our model’s predictive ability to single time point alternatives: the CCI, the SAS, the ASA-PS, the Elixhauser measure, Quan’s CCI and Elixhauser variants, the Risk Stratification Index (RSI), the Risk Quantification Index (RQI), Le Manach’s Perioperative Score to Predict Postoperative Mortality (POSPOM), and the Surgical Mortality Probability Model (S-MPM) [3, 7, 14, 19–25].

## Methods

Both the University of Texas MD Anderson Cancer Center and Rice University Institutional Review Boards approved this research and waived the need for informed patient consent.

### I Study Population

This research was based on 81,196 surgical patients treated at MD Anderson between January 2007 and March 2014. MD Anderson is a Comprehensive Cancer Center located in Houston, Texas. We studied adult patients undergoing major abdominal surgery, including all major procedures below the diaphragm and above the pelvic floor, whether intraperitoneal or extraperitoneal. 2,791 cases involved multiple procedures. Our data sources were administrative billing and procedure codes as well as surgical data from the Anesthesia Information Management System and the hospital’s nightly census.

### II Study Design

**Figure 1:**
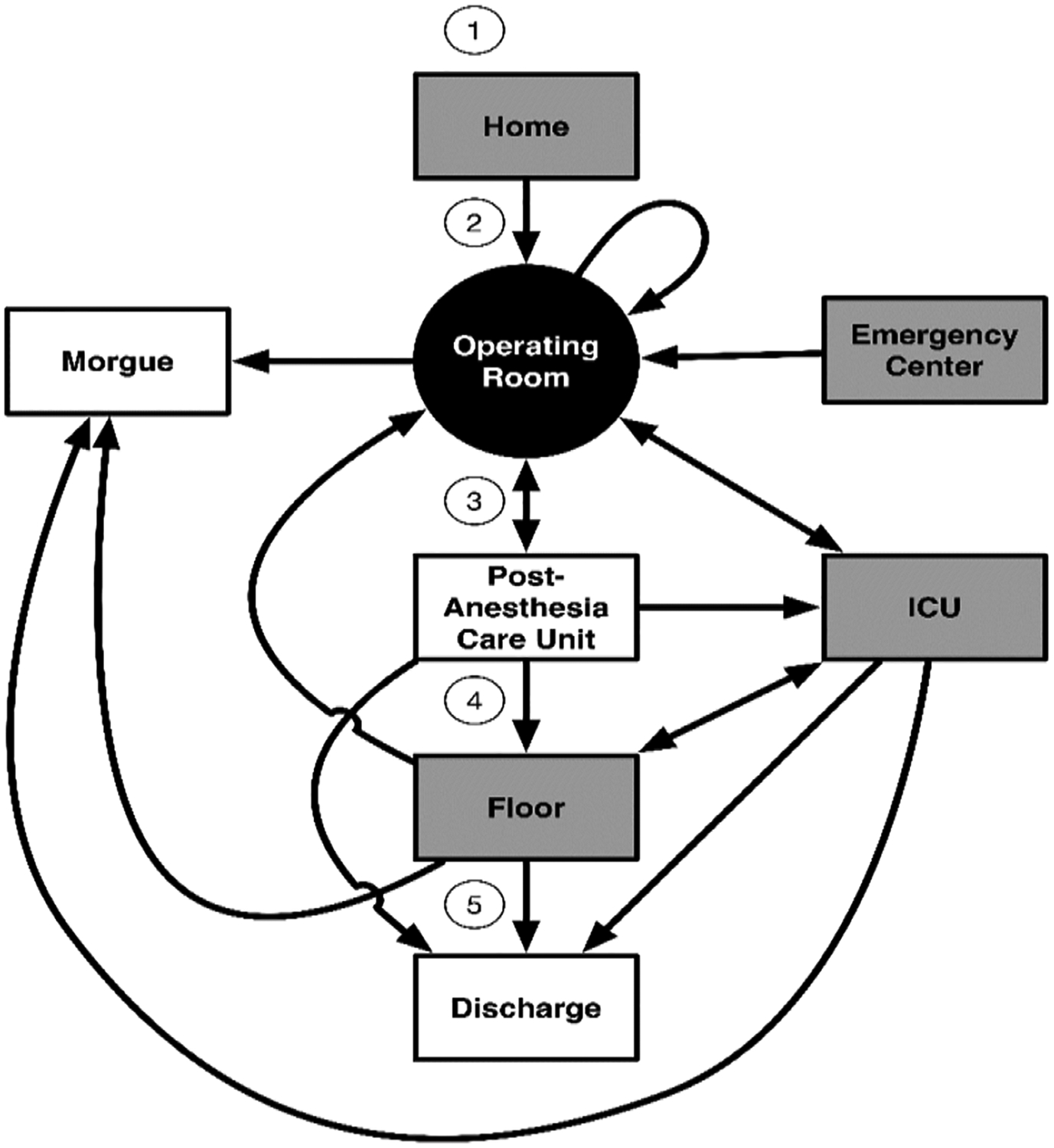
Relevant perioperative patient course through the hospital. Shaded boxes are starting locations.

We built models of 30- and 90-day mortality for cancer patients undergoing major abdominal surgery that improves in predictive ability over time as we learn more about each patient. We started with demographic information and added features as the patient arrived for surgery and progressed through the operating theater and perioperative stay until the sixth postoperative day. At each point, we added the newly available features to the cumulative model and re-predicted. Figure 1 shows the points of interest around the perioperative period. In this diagram, for example, a patient arrived from home on the morning of surgery (1), had the operation (2), recovered for a few hours in the PACU (3), and then was admitted to a ward for further recovery and observation (4). One week later, the patient returned home (5).

**Figure 2:**
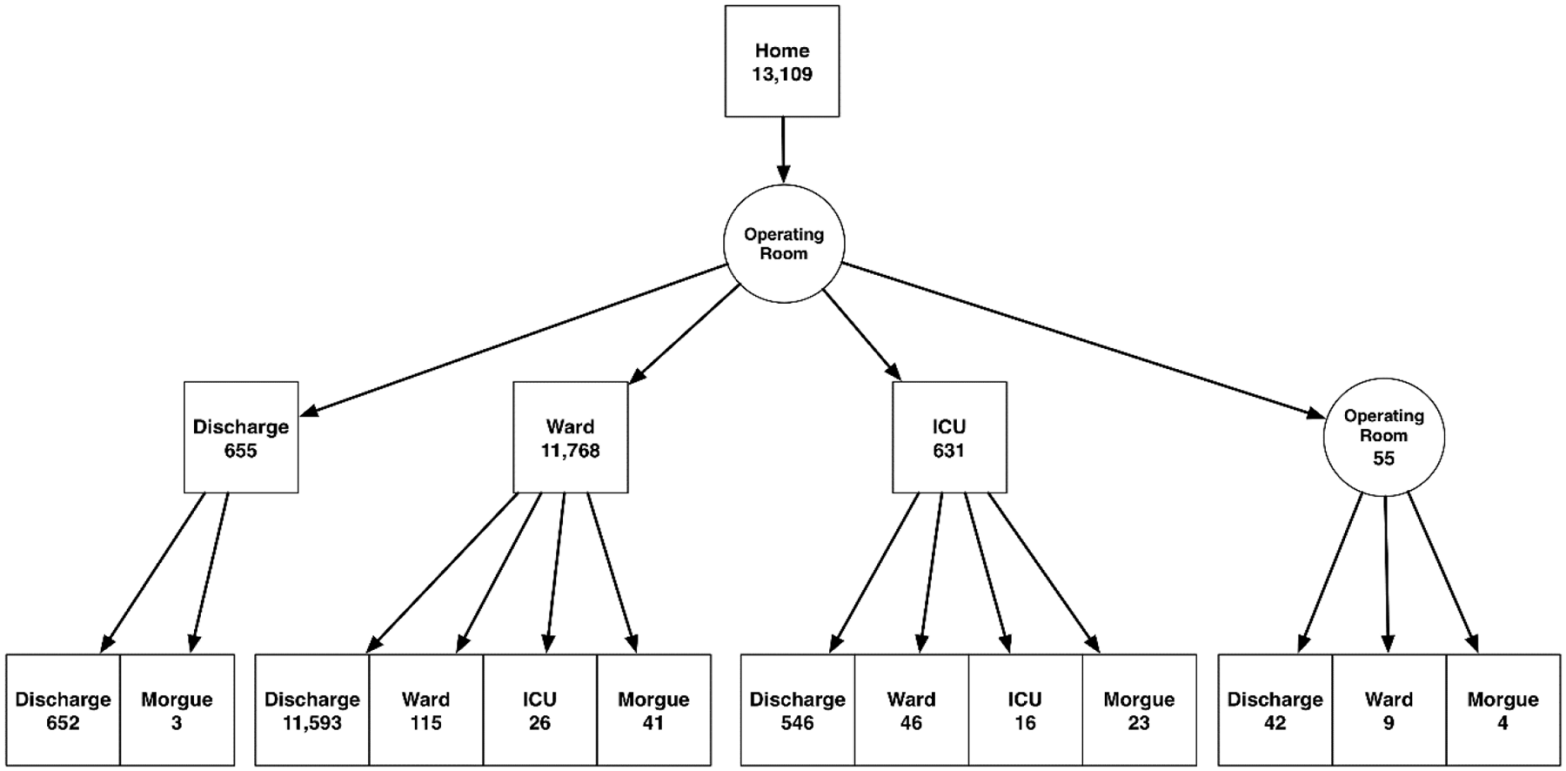
Destinations and quantities of abdominal surgery patients who started at home. The bottom row shows the patients’ 30-day location.

We considered four different starting points for the cases, as shown in (Figure 1). These locations include arrivals from home or via the emergency center and two inpatient locations, the ICU and the hospital ward. From these starting points, the patients proceeded to the operating room, and then either returned immediately to surgery, were sent to the ICU, went to the PACU or were sent to the morgue. In our dataset, only one patient died during surgery. Figure 2 shows the number of patients who started at home on the day of surgery, the patients’ surgical destination and 30 (90)-day status. The supplemental material includes analogous diagrams starting from the Emergency Center, hospital ward and ICU.

### III Incremental Steps and Features

Rather than rely on a single time point, we evaluated patients as they went through the “process of care” and leveraged that information to build a cumulative perioperative model to identify patients with higher mortality risk. The time points during the patient’s clinical course were as follows:

- When surgery is scheduled
- The morning of surgery
- When the procedures are complete
- When anesthesia is complete
- After leaving the PACU
- Six days postoperative

We considered a number of patient demographic and case data for our predictive model, as listed in (Table 1). We determined procedures performed from CPT codes recorded in the electronic health record and comorbidities from ICD-9 codes.

At the time surgery is scheduled, we know the patient’s age, gender, race, comorbidities and the scheduled procedure. To reflect each patient’s comorbidities, we used the CCI [3]. The CCI is a weighted sum of 19 key comorbidities, each assigned a weight of 1, 2, 3 or 6 [3]. For consistency, we use Deyo’s mapping of the comorbidities to ICD-9 codes [26]. By the morning of surgery, we know the patient’s ASA-PS classification, whether or not the surgery is emergent, and where the patient was prior to surgery (home, on an inpatient ward, in the ICU, or in the emergency center).

We took two steps to model what occurred in the operating room. First, we looked at which abdominal procedures were performed, and once anesthesia ended, we included the patient’s SAS. Next, we incorporated the patient’s surgical destination (discharge, inpatient ward, ICU, return to surgery). Finally, we looked forward to six days to identify the patient’s location (home, ICU, institutional care, etc.). We discarded features that either had no discrimination (e.g., whether or not the patient had a splenectomy) or if the feature did not improve the C-statistic for the step (e.g., extended PACU stays).

### IV Outcome Measures

We evaluated our model’s ability to predict 30 and 90-day mortality using the C-statistic, or area under the receiver operating characteristic curve (AUROC). This metric reflects a model’s discriminative ability, with a value of 0.5 equivalent to a random or coin-flip classifier, and a value of 1 indicating a perfect ability to segregate positive cases from negative. Mortality was determined either by discharge status within 30 (90) days or by records indicating the date of last contact and status. In addition, the presence of any patient information past 30 (90) days postoperative, indicated survival.

### V Comparisons with Other Predicting Models

We implemented the time point assessment scores as described in their respective papers, with a few exceptions. Since we did not have access to the planned surgical procedures, we used the actual procedure performed in the POSPOM model. With regard to the S-MPM model, not all of the procedures in our study were included in the S-MPM model. Therefore, we assigned the missing procedures to high, intermediate, or low risk categories based on our medical expertise and judgment. We also changed the categorization of pelvic exenteration from low-risk (all gynecologic procedures) to high-risk, based on how this procedure is performed at MD Anderson. We compared our models’ predictive ability to single time point alternatives: the CCI, the SAS, the ASA-PS, the Elixhauser measure, Quan’s CCI and Elixhauser variants, RQI, RSI, the POSPOM, and the S-MPM [3, 7, 14, 19–25].

The RQI composite major morbidity/mortality risk estimate includes a Procedure Severity Score for mortality, ASA-PS, and hospitalization type (in or out-patient) [23]. To calculate the RQI in cases with multiple procedures, we selected the procedure with the highest weight. The Risk Stratification Index (RSI) uses procedure and diagnosis codes to predict a patient’s hospital length of stay and mortality [22]. Both the RQI and the RSI predict 30-day mortality and cover a broad spectrum of patients and conditions. The S-MPM categorizes surgeries into three tiers based on risk and utilizes the ASA-PS and emergency status of the operation [25]. The S-MPM was developed using 298,772 patients undergoing noncardiac surgery from the National Surgical Quality Improvement Program (NSQIP). SAS, a 10-point score that predicts 30-day mortality and major surgical complications, was developed and validated on both colectomy and general and vascular procedures use estimated blood loss, lowest heart rate and lowest surgical mean arterial pressure [14].

Combining available patient information prior to the procedure, Le Manach *et al.*’s POSPOM score uses demographics and comorbidities in conjunction with the surgery category [24]. While the POSPOM score was intended to predict in-hospital mortality, we used it to predict 30 and 90-day mortality.

### VI Statistical Methods

Two-thirds of the patients (9,251) were randomly assigned to the training cohort and the remaining (4,625) to evaluation. The predictive model was built using the training cohort and evaluated on the evaluation cohort. Our model is a multivariate logistic regression model. In the first step, we selected the demographic and comorbidity features that, combined, produced the highest C-statistic. Afterward, we added discriminative features by the time available, using forward feature selection, retaining any discriminating candidate feature that increased the C-statistic.

Prior to computing the SAS, we removed outlier values from the vital sign data. We treated heart rates less than 40 beats per minute as artifact and calculated the mean blood pressure (MBP) from systolic (SBP) and diastolic (DBP) values.

$$\left(MBP=\frac{1}{3}\times \left(2\times DBP+SBP\right)\right)$$

We required the SBP to be at least 40 mmHg and the diastolic to be at least 28 mm Hg. Furthermore, we required a minimum 12-point difference between the two readings. In an effort to simplify the CPM and improve predictability, we also reduced the 10-point scale to the top six values. Our final values for the surgical Apgar score are less than 5, 6, 7, 8, 9 and 10. Finally, at each time step, we remove all patients in the evaluation set that have died. We computed confidence intervals using standard error calculations, as proposed by Hanley and McNeil [27].

## Results

Overall, there was a 0.76% (2.09%) 30 (90)-day mortality rate for these cases. The patients’ median SAS was 7, and 6% of patients were in the post-anesthesia care unit (PACU) overnight. Typical postoperative lengths of stay were 7.6 days, and 2% of patients were admitted to the ICU after first being in an inpatient ward. Patients were typically in their late sixties, with a fairly even mix of men and women. Over 98% of the cases were elective, with over 94% of the patients arriving from home the day of surgery. Approximately 5% of the patients were already inpatients, and a small number (~0.5%) arrived at surgery via the hospital emergency center. Table 1 shows our patients’ characteristics. Table 2 lists the exact feature values used in the models and the univariate C-statistic for each candidate feature. For the 30-day CPM model, the most significant independent predictors of outcome were the CCI (0.69), SAS (0.76), and surgical destination (0.72).

**Figure 3:**
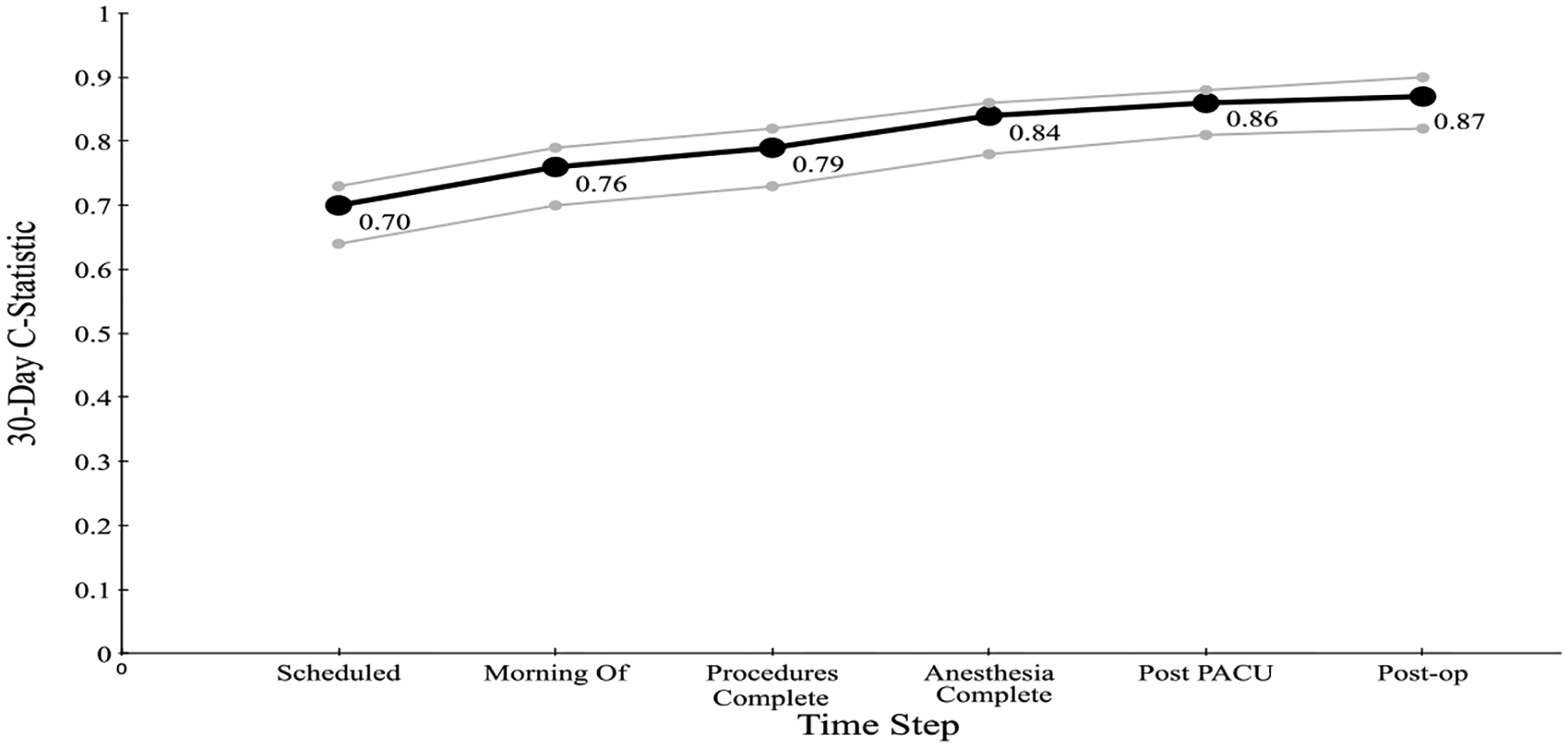
C-statistic for the Cumulative Perioperative Model Predicting 30-Day Mortality, with Confidence Intervals.

**Figure 4:**
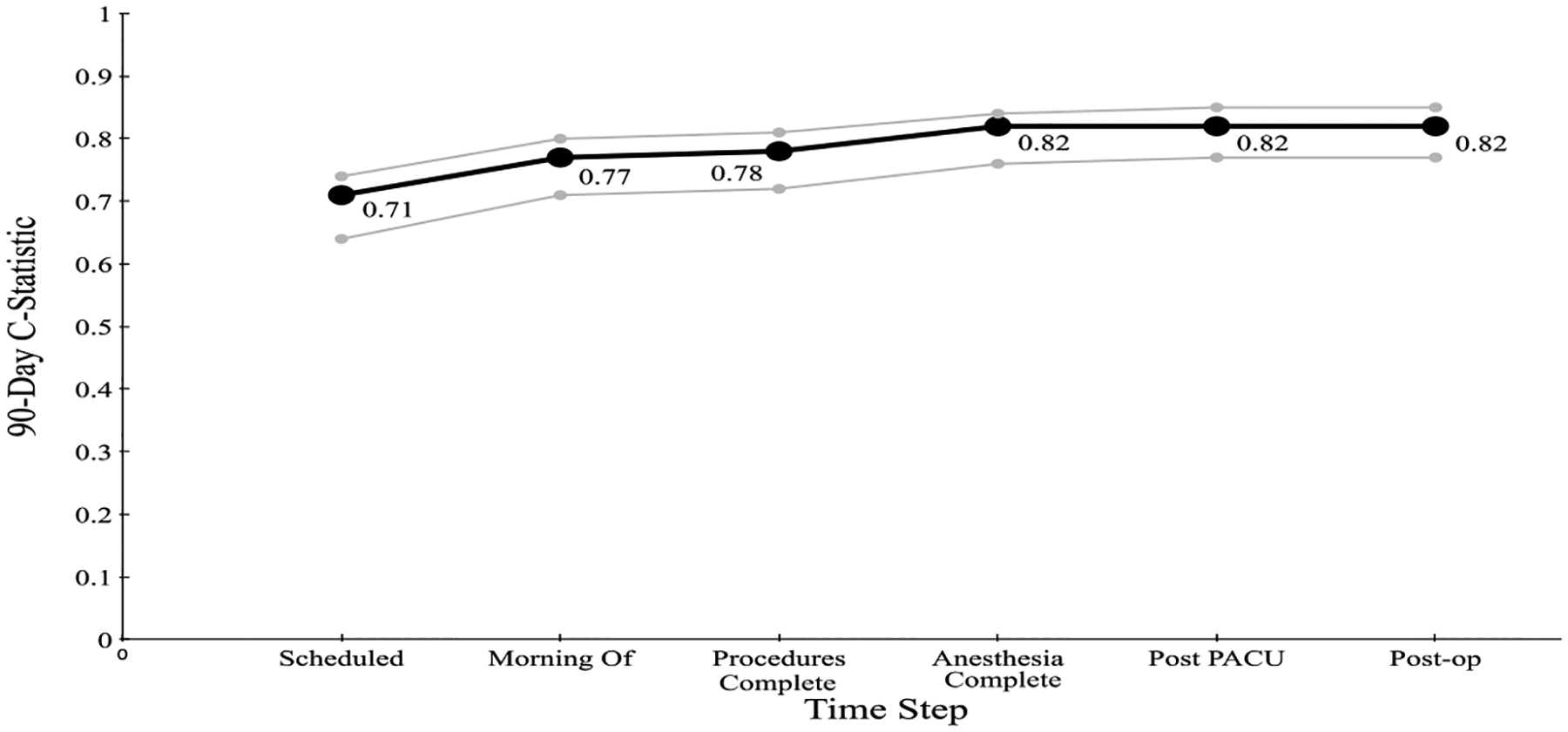
C-statistic for the Cumulative Perioperative Model Predicting 90-Day Mortality, with Confidence Intervals.

Figures 3 & 4 show the C-statistic for predicting (30 and 90-day) mortality generated by our model at each time step: when the case is scheduled; the morning of the surgery; after procedure completion; at anesthesia end; post PACU, and six days postoperatively. The 30-day C-statistic starts with a value of 0.70 and increases, basically linearly, as time progresses, to a value of 0.87.

Table 3 shows the results from the point-in-time models as well as the CPM model at each point in time. As can be seen, the C-statistic improves over time and outperforms the other models at each step.

## Discussion

In this study, we confirmed the hypothesis that by adding time-based features to a cumulative model, we are better able to predict 30 (90)-day mortality in cancer patients undergoing abdominal surgery. The 30-day mortality rate is only 0.80% in our evaluation set, which makes this prediction problem difficult. Given this prevalence, obtaining a C-statistic of 0.70 (0.71) before surgery begins, 0.84 (0.82) after anesthesia, and 0.86 (0.82) post PACU is quite remarkable, especially considering the small and easily obtainable features required [28]. Additionally, the model performed well above the other ten other available models starting the morning of surgery and better than the other models postoperatively.

A number of predictive scores have been developed specifically for the surgical population. Once the procedure is scheduled, comorbidity information is available, which may be used for computing mortality scores. The foremost comorbidity indexes are the CCI and the Elixhauser score [3, 20]. While originally developed for longer-term forecasts, the CCI is commonly used to assess a patient’s mortality risk [29, 30]. Since their formation, numerous updates and variants of the CCI and Elixhauser scores have been implemented [26, 31–34]. We considered using the Elixhauser score measures in lieu of the CCI, as they are based on a larger population and frequently outperform the CCI [35–37].

However, the CCI is simpler to compute, as there are no rules to follow regarding which comorbidities to include. In addition, the CCI reduces the comorbidities to a single score instead of the up to 30 features used by the Elixhauser score [3, 20]. Instead, we compare the use of the Elixhauser score to predict 30 and 90-day mortality independently and compare this model with the CPM. The ASA-PS has been shown prospectively to strongly correlate with surgical outcomes, including mortality, with higher scores associated with higher incidences of poor outcomes [38].

Using the CPM alone, we obtained a C-statistic of 0.87 for predicting 30-day mortality, on par with the most predictive individual features in our model. Since the RQI uses the patient’s primary procedure, it is most likely better suited to a patient population where only one procedure is performed at a time. In our cohort, over 20% of the cases had multiple procedures. Using the covariates provided by Sessler *et al*. (RSI), we calculated the C-statistic for in-hospital mortality (coefficients were not available for 30-day mortality) and obtained a C-statistic of 0.58 using our evaluation data. This value is significantly less predictive than using the CCI alone on our patients. While the RSI model is based on over 17 million Medicare patients, it excludes patients younger than 65 and requires the use of many different codes, consisting of 187 regression coefficients. It performed reasonably well on our patient cohort but did not outperform key individual predictors.

Both the RSI and RQI were designed for large, heterogeneous populations. They use a significant number of diagnosis and/or procedure codes, often with subtle differences and weights. Conversely, the CPM includes commonly available and easily computable patient information. For example, we require a simple Yes/No answer to whether or not one of six key procedures (represented by 36 CPT codes present in our dataset) was performed. The most complex calculations for the CPM are the CCI and the SAS. Patients in our cohort are very similar in terms of the feature values used by the RSI and RQI models, driving the need for a more specific model such as the CPM, which is better able to differentiate patients.

The most important features contributing to poor prognosis in both models are starting in the EC or ICU, emergent status (90-day), and being in the ICU six days postoperatively (30-day). The most significant features indicating better prognosis are starting from home on the day of surgery and going to the hospital ward after surgery. These findings, while intuitive, enable earlier and more accurate predictions of outcomes based on specific patient characteristics within a cohort of similar patients. These model-based predictions can be the starting point for engaging the patient and families in the decision-making process during discussions about prognosis or progression of disease and could help physicians review and impact clinical courses and outcomes for similar patients. This risk assessment could be used at every step along the way to support clinical decisions or adjust the level of patient care and oversight.

Our study does have limitations. It is retrospective and is a single-site study which limits the generalizability to other centers. Further, we may be missing information on follow-up care for some patients as MD Anderson often treats visiting patients. Missing follow-ups may result in inaccuracies in the mortality count. However, since the surgeries considered are major, we expect a relatively high level of follow-up. In addition, many of the predictive models were not intended to predict 30-or 90-day mortality but were tuned to predict in-hospital mortality (POSPOM) or included significant morbidities as well. We did not address how the information might affect clinical decision making [39].

Finally, we are planning to further refine the model using more sophisticated techniques and additional features to increase the predictive ability sooner, providing earlier insight into patients at risk. There are additional features that we believe would add to the predictive power of this model, in particular, primary diagnosis and disease stage, to allow adjustments for cancer progression. Once the patient reaches the ICU, adding the APACHE and/or SOFA scores are likely to improve predictions. We would also like to incorporate the discharge destination and predict additional complications (e.g., acute coronary syndrome, stroke).

## Conclusion

We have developed a cumulative perioperative model (CPM) that reevaluates a patient’s state over time. This model increases in predictive capability as we follow patients through the hospital. The CPM demonstrates the value of using time-dependent information from the patient’s perioperative clinical course and could be used to identify patients who would benefit from different treatments or postoperative levels of monitoring. This tool enables an earlier assessment of patient risk, which could then be used as a decision support aid regarding care and treatment, potentially resulting in improved outcomes, decreased costs, and more informed decisions.

## Summary

Prior to this research, the following was known: Single point in time risk scores can be used to predict a patient’s complication and/or mortality risk.

This study added to our knowledge: Continuous models that incorporate additional information throughout a patient’s surgical course increase the ability to predict mortality risk for cancer patients undergoing abdominal surgery.

## Supplementary Material

Supplementary Material

## Figures and Tables

**Table 1:** Major Abdominal Surgery patient characteristics.

Characteristic	Training	Evaluation	Total
Number of cases	9,251	4,626	13,877
30-day mortalities # / %	69 / 0.75%	37 / 0.80%	106 / 0.76%
90-day mortalities # / %	204 / 2.21%	86 / 1.86%	290 / 2.09%
Age, median (IQR), years	59.9(43.4, 63.8)	59.8(43.3, 63.9)	59.9(43.3, 63.8)
Male gender, %	45	47	46
Race/ethnicity, %			
White	73.2	73.4	73.3
Black	7.8	7.9	7.9
Hispanic	13.2	13.1	13.2
Other	5.8	5.6	5.7
BMI			
< 18.5	1.4%	1.6%	1.4%
>= 18.5, < 25	26.4%	26.7%	26.5%
>= 25, < 30	34.0%	33.0%	33.6%
>= 30, < 35	21.4%	22.6%	21.8%
>= 35, < 40	9.4%	9.3%	9.4%
> 40	7.4%	6.9%	7.2%
Charlson Index, median (IQR)	5 (2,8)	5 (2,8)	5 (2,8)
Start Location, %			
Home	94.3	94.7	94.5
Ward	5.0	4.6	4.9
EC	0.5	0.4	0.5
ICU	0.2	0.3	0.2
Emergency Status, %	1.1	1.0	1.1
ASA Score, median, (IQR)	3 (1,3)	3 (1,3)	3 (1,3)
Scheduled admit type %			
Out-patient	1.5	1.8	1.6
Observation Unit	1.3	1.4	1.4
Same Day Admit	91.9	91.7	91.8
In-patient	5.2	5.0	5.1
Unknown	0.1	0	0.1
Presurgical LOS, median (IQR), years	0 (0,0)	0 (0,0)	0 (0,0)
Procedures, # / % of total procedures			
Nephrectomy	2,024	1,009	3,033 / 19.4%
Colectomy	2,000	938	2,938 / 18.8%
Hysterectomy	1,644	781	2,425 / 15.5%
Hepatectomy	1,088	539	1,627 / 10.4%
Cystectomy	862	455	1,317 / 8.4%
Pancreatectomy	676	331	1,007 / 6.5%
Enterectomy	501	259	760 / 4.9%
Oophorectomy	394	207	601 / 3.9%
Splenectomy	260	119	379 / 2.4%
Jejunostomy	208	129	337 / 2.2%
Enterostomy	201	120	321 / 2.1%
Adrenalectomy	211	99	310 / 2.0%
Gastrectomy	190	95	290 / 1.9%
Pelvic exenteration	100	70	170 / 1.1%
Enteroenterostomy	56	32	88 / 0.6%
Surgical Apgar score, median (IQR)	7 (5,7)	7(5,7)	7 (5,7)
Extended PACU stay, %	6.4	6.0	6.3
Patient Location on Day 7			
Home	5024	2507	7531 / 54.3%
Home Care	193	90	283 / 2.0%
Institutional Care	14	6	20 / 0.1%
PACU	9	3	12 / 0.1%
Hospital Ward	3833	1915	5748 / 41.4%
ICU	166	97	263 / 1.9%
Hospice	0	0	0 / 0 %
Morgue	12	8	20 / 0.1%
Post-operative Length of stay, median (days)	7.7	7.6	7.7

**Table 2:** Discrete Feature Values used in the Cumulative Perioperative Model associated with each cumulative step and their univariate ability to predict mortality.

Time step	Feature	Values	C-statistic for 30-day mortality	Cumulative C-statistic for 30-day mortality	C-statistic for 90-day mortality	Cumulative C-statistic for 90-day mortality
1. Procedure Scheduled	Gender	{Female, Male}	0.56	-	0.56	-
	Race	{White, Black, Hispanic, Other}	0.58	-	0.49	-
	Age	numeric	0.58	-	0.60	-
	BMI	6 groups	0.46		0.55	
	Charlson Comorbidity Index	Discrete values from 0 – 20, inclusive	0.69		0.69	
Best step 1 features	Race + CCI			0.70	Gender + CCI	0.71
2. Morning of surgery	Start location	{Home, Ward, ICU, EC}	0.61		0.61	
	Emergency status	{Absent, Present}	0.58		0.57	
	ASA classification	Numeric: {1,2,3,4,5}	0.65		0.62	
	Best step 1 & all step 2 features			0.76		0.78
3. Procedures complete	Adrenalectomy	{Absent, Present}	0.49	Excluded	0.49	Excluded
	Colectomy	{Absent, Present}	0.48	Excluded	0.52	
	Cystectomy	{Absent, Present}	0.54		0.53	
	Enterectomy	{Absent, Present}	0.57		0.55	
	Enteroenterosto my	{Absent, Present}	0.51		0.52	
	Enterostomy	{Absent, Present}	0.53		0.53	
	Gastrectomy	{Absent, Present}	0.50	Excluded	0.49	Excluded
	Hepatectomy	{Absent, Present}	0.48	Excluded	0.52	
	Hysterectomy	{Absent, Present}	0.57		0.57	
	Jejunostomy	{Absent, Present}	0.51		0.51	
	Nephrectomy	{Absent, Present}	0.48	Excluded	0.51	
	Oophorectomy	{Absent, Present}	0.50	Excluded	0.51	
	Pancreatectomy	{Absent, Present}	0.52		0.51	
	Pelvic exenteration	{Absent, Present}	0.51		0.51	
	Splenectomy	{Absent, Present}	0.50	Excluded	0.50	Excluded
	Best step 1, all step 2, & discriminative step 3 features			0.79		0.78
4. Anesthesia complete	Surgical Apgar score	{≤5, 6, 7, 8, 9, 10}	0.76	0.84	0.70	0.82
5. Post PACU	Extended PACU	{Absent, Present}	0.52	Excluded	0.52	Excluded
	Surgical destination	{Discharge, Ward, ICU, Surgery}	0.72	0.86	0.63	0.82
6. Six days post-operative	Delayed ICU admit	{Home, Home Care, Institutional Care, PACU, Hospital Ward, ICU}	0.56	0.87	0.53	0.84

**Table 3:** Comparison of 30-day and 90-day mortality predictions by all models, in time order.

Model	30-day Mortality	90-day Mortality
1. Procedure Scheduled		
CPM	0.70	0.71
CCI	0.69	0.69
Quan CCI	0.71	0.68
Elixhauser	0.71	0.73
Quan Elixhauser	0.68	0.69
2. Morning of surgery		
CPM	0.76	0.78
ASA	0.65	0.62
POSPOM	0.63	0.65
3. Procedures complete		
CPM	0.79	0.78
S-MPM	0.72	0.68
RSI	0.58	0.67
RQI	0.70	0.71
4. Anesthesia complete		
CPM	0.84	0.82
SAS	0.76	0.70
