# Supplementary material for: The Cumulative Perioperative Model: Predicting 30-Day Mortality in Abdominal Surgery Cancer Patients
Source: J Surg Oncol (Tallinn). Author manuscript; Available in PMC 2021 Oct 7. (PMC8496410; doi:10.31487/j.jso.2020.01.10)
Supplement: Supplementary Material [file NIHMS1578530-supplement-Supplementary_Material.pdf]

Available online at www.sciencerepository.org

Science Repository

Supplementary Material

The Cumulative Perioperative Model: Predicting 30-Day Mortality in Abdominal Surgery Cancer Patients

Risa B Myers[1,2], Joseph R Ruiz[3], Christopher M Jermaine[1] and Joseph L Nates[4*]

[1]*Department of Computer Science, Rice University, Texas, USA*
[2]*Children's Environmental Health Initiative, Rice University, Texas, USA*
[3]*Department of Anesthesiology and Perioperative Medicine, Division of Anesthesiology and Critical Care, University of Texas MD Anderson Cancer Center, Texas, USA*
[4]*Department of Critical Care, Division of Anesthesiology and Critical Care, University of Texas MD Anderson Cancer Center, Texas, USA*

ARTICLE INFO

Article history:
Received: 17 February, 2020
Accepted: 2 March, 2020
Published: NA

Keywords:
Regression analysis
perioperative period
mortality
decision support techniques

ABSTRACT

Objectives: 1) To develop a cumulative perioperative model (CPM) using the hospital clinical course of abdominal surgery cancer patients that predicts 30 and 90-day mortality risk; 2) To compare the predictive ability of this model to ten existing other models.

Materials and Methods: We constructed a multivariate logistic regression model of 30 (90)-day mortality, which occurred in 106 (290) of the cases, using 13,877 major abdominal surgical cases performed at the University of Texas MD Anderson Cancer Center from January 2007 to March 2014. The model includes race, starting location (home, inpatient ward, intensive care unit or emergency center), Charlson Comorbidity Index, emergency status, ASA-PS classification, procedure, surgical Apgar score, destination after surgery (hospital ward location) and delayed intensive care unit admit within six days. We computed and compared the model mortality prediction ability (C-statistic) as we accumulated features over time.

Results: We were able to predict 30 (90)-day mortality with C-statistics from 0.70 (0.71) initially to 0.87 (0.84) within six days postoperatively.

Conclusion: We achieved a high level of model discrimination. The CPM enables a continuous cumulative assessment of the patient's mortality risk, which could then be used as a decision support aid regarding patient care and treatment, potentially resulting in improved outcomes, decreased costs and more informed decisions.

© 2020 Joseph L Nates. Hosting by Science Repository. All rights reserved.

Correspondence to: Joseph L Nates, M.D., M.B.A., Department of Critical Care, Division of Anesthesiology and Critical Care, University of Texas MD Anderson Cancer Center, 1515 Holcombe Blvd Unit #112, Houston, Texas 77030, USA; Tel: 7137925040; Fax: 7137451869; E-mail: jlnates@mdanderson.org

© 2020 Joseph L Nates. *This is an open-access article distributed under the terms of the Creative Commons Attribution License, which permits unrestricted use, distribution, and reproduction in any medium, provided the original author and source are credited.* Hosting by Science Repository. All rights reserved.
http://dx.doi.org/10.31487/j.JSO.2020.01.10.SUP

Diagrams illustrating patient starting and 30-day locations

Figures A - D, below, show the starting and ending points of patients' clinical courses, starting from home, the emergency center and the ICU.

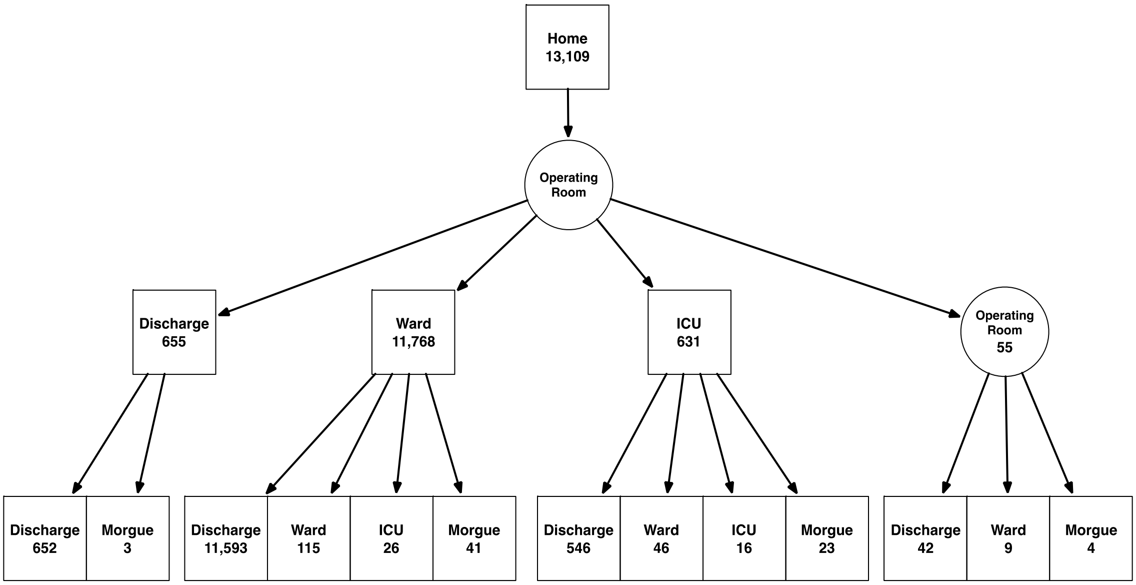

Figure SA: Destinations and quantities of abdominal surgery patients who started at home. The bottom row shows the patients' 30-day location.

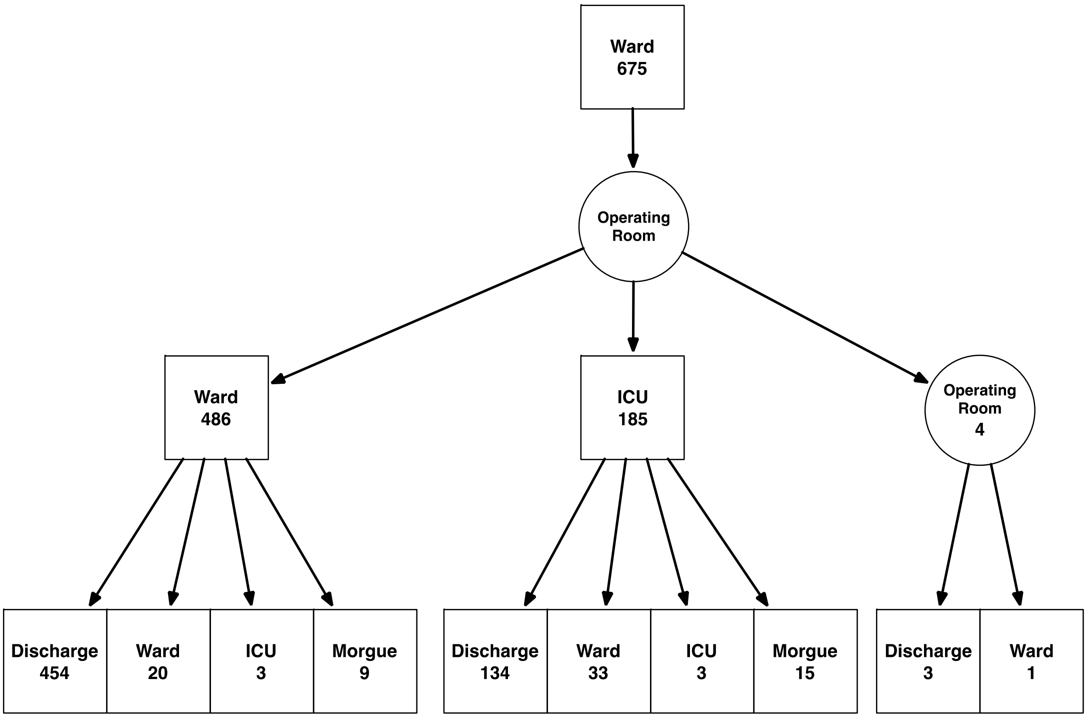

Figure SB: Destinations and quantities of abdominal surgery patients who started as non-intensive care unit inpatients. The bottom row shows the patients' 30-day location.

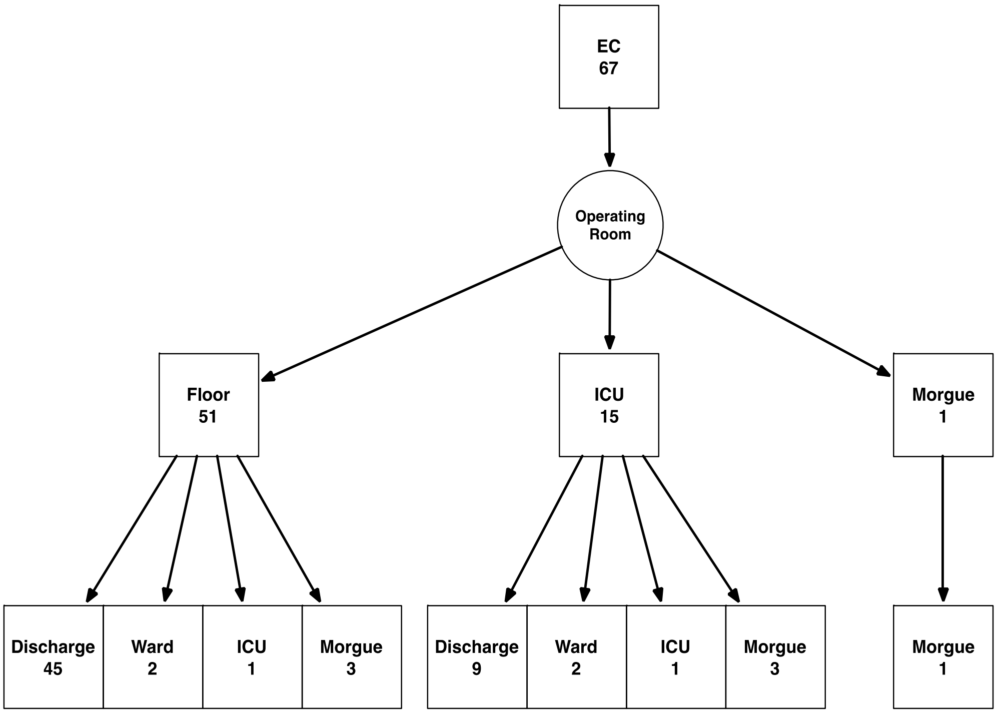

Figure SC: Destinations and quantities of abdominal surgery patients who started in the emergency center. The bottom row shows the patients' 30-day location.

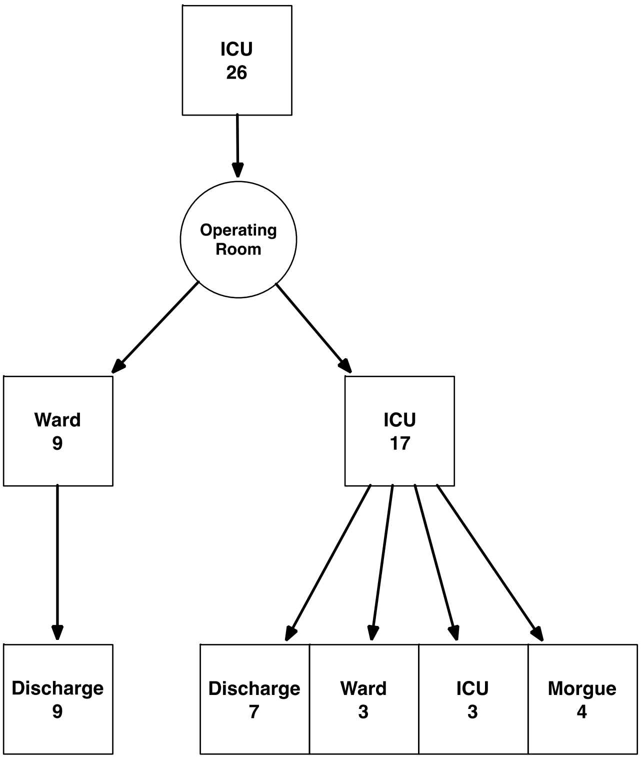

Figure SD: Destinations and quantities of abdominal surgery patients who started in the hospital intensive care unit. The bottom row shows the patients' 30-day location.
